# Inhibition of *Pseudomonas aeruginosa* LPS‐Induced airway inflammation by RIPK3 in human airway

**DOI:** 10.1111/jcmm.17579

**Published:** 2022-10-13

**Authors:** Minsu Yun, Sun‐Hee Park, Dong Hee Kang, Ji Wook Kim, Ju Deok Kim, Siejeong Ryu, Jeongyeob Lee, Hye Min Jeong, Hye Ran Hwang, Kyoung Seob Song

**Affiliations:** ^1^ Department of Anesthesiology and Pain Medicine Kosin University College of Medicine Busan South Korea; ^2^ Department of Medical Science Kosin University College of Medicine Busan South Korea

**Keywords:** histone, LPS, pro‐inflammatory cytokine, *Pseudomonas aeruginosa*, RIPK3, ubiquitination

## Abstract

Although the physiological function of receptor‐interacting protein kinase (RIPK) 3 has emerged as a critical mediator of programmed necrosis/necroptosis, the intracellular role it plays as an attenuator in human lungs and human bronchial epithelia remains unclear. Here, we show that the expression of RIPK3 dramatically decreased in the inflamed tissues of human lungs, and moved from the nucleus to the cytoplasm. The overexpression of RIPK3 dramatically increased F‐actin formation and decreased the expression of genes for pro‐inflammatory cytokines (IL‐6 and IL‐1β), but not siRNA‐RIPK3. Interestingly, whereas RIPK3 was bound to histone 1b without LPS stimulation, the interaction between them was disrupted after 15 min of LPS treatment. Histone methylation could not maintain the binding of RIPK3 and activated movement towards the cytoplasm. In the cytoplasm, overexpressed RIPK3 continuously attenuated pro‐inflammatory cytokine gene expression by inhibiting NF‐κB activation, preventing the progression of inflammation during *Pseudomonas aeruginosa* infection. Our data indicated that RIPK3 is critical for the regulation of the LPS‐induced inflammatory microenvironment. Therefore, we suggest that RIPK3 is a potential therapeutic candidate for bacterial infection‐induced pulmonary inflammation.

## 1 INTRODUCTION

*Pseudomonas aeruginosa* is a Gram‐negative bacterial pathogen related to a wide range of infections including lung disease.^1^, ^2^ Because of its metabolic flexibility and fundamental resistance to antimicrobials, *P*. *aeruginosa* grows well in a wide variety of materials and environments, including in‐hospital facilities and patient devices.^3^ Although it rarely infects healthy individuals, it is a leading and well‐known opportunistic pathogen, especially in immunocompromised patients with defective immune defences.^4^ *P. aeruginosa* is known to colonize and infect the lungs of patients with cystic fibrosis (CF) and advanced stages of chronic obstructive pulmonary disease (COPD).^5^, ^6^ Most importantly, *P. aeruginosa* has multiple antibiotic resistance and tolerance that allow it to survive antibiotic treatment against infection.^1^ Moreover, bacterial coinfection is a mutual complication of several viral pulmonary tract infections, such as severe acute respiratory syndrome coronavirus 2 (SARS‐CoV‐2), and significantly increases morbidity and mortality.^7^ Therefore, a new therapeutic strategy is needed for antibiotic‐dependent microbial treatment.

Receptor‐interacting serine/threonine‐protein kinase (RIPK) 3 is a major protein of necroptosis that can be initiated by various signals including the activation of death receptors, Toll‐like receptors, interferon receptors and pathogen infection.^8^, ^9^ Necroptosis is a system of regulated cell death characterized by necrotic features including cell swelling and disrupted cell plasma membrane.^10^ Necroptosis is tightly regulated by the activation of RIPK1 and RIPK3.^10^ RIPK3 is regulated by the proteins related to post‐translational modification, such as protein phosphatase 1B,^11^ deubiquitylating enzyme A20,^12^ C terminus of Hsp‐70‐interacting protein (CHIP)^13^ and Pellino E3 ubiquitin protein ligase 1 (PELI1).^14^ Although RIPK3 is a pivotal protein in necroptosis, post‐translational processes that may control RIPK3 activity and stability during *P. aeruginosa* infection in the airway remain poorly understood.

Disruption of cell‐to‐cell barriers is a major feature of tissue inflammation.^15^ An excessive increase in epithelial permeability has critically harmful effects on tissue homeostasis by increasing body exposure to detrimental environmental agents and inducing uncontrollable inflammation.^16^, ^17^, ^18^ Therefore, actin remodelling plays a very important role in maintaining cell homeostasis by suppressing pathogen infection and controlling inflammation. The biochemical and physiological mechanism by which actin remodelling and trans‐epithelial electrical resistance (TEER) may be affected by RIPK3 during *P. aeruginosa* infection in respiratory diseases remains unclear.

This study aims to characterize whether the RIPK3 protein has an anti‐inflammatory effect against *P. aeruginosa* in the airway. Overexpression of RIPK3 could down‐regulate LPS‐induced inflammation, but not siRNA‐RIPK3. Interestingly, the RIPK3 protein can bind histone to inhibit translocalization into the cytoplasm. After the methylation of histone with LPS stimulation, RIPK3 translocates into the cytoplasm to decrease the production of pro‐inflammatory cytokines dramatically. Our data indicate that the RIPK3 protein is critical for the regulation of the LPS‐induced inflammatory microenvironment in respiratory diseases.

## 2 MATERIALS AND METHODS

### 2.1 Lung tissue array

A human lung disease spectrum tissue array was purchased from US Biomax (Rockville). The tissue array consisted of 24 cases (48 cores), which included normal lung tissue, lung hyperplasia of stroma, and pulmonary fibrosis with chronic inflammation of bronchiole, lung lobar pneumonia, pulmonary atelectasis, collapsed lung, pulmonary tuberculosis, pulmonary emphysema, and inflammatory pseudotumor plus lung small cell carcinoma, lung adenocarcinomas, and lung squamous cell carcinomas. Of these, we used three different groups of tissues: normal, hyperplasia and inflammation. Lung sections were processed with either anti‐RIPK3 antibody, phosphor‐RIPK3 antibody (Ser277) or goat anti‐rabbit IgG (Alexa Fluor 546), stained with Deep‐Red, and images were taken for each core. Five separate images of each section were given a score^1^, ^2^, ^3^, ^4^, ^5^, ^6^, ^7^, ^8^, ^9^, ^10^ by three independent scientists.

### 2.2 Materials and Cell Culture

The LPS was purchased from Merck. Purified cytokines and specific ELISA kits were purchased from R&D Systems. Normal human bronchial epithelial (BEAS‐2b) cells were purchased from ATCC, and cells were cultured in BEBM (Lonza) with a BEGM kit at 37°C in a humidified incubator with 5% CO_2_. The culture plates were pre‐coated with a mixture of 0.01 mg/ml fibronectin (Sigma‐Aldrich), 2 mg/ml gelatin (Sigma‐Aldrich) and 0.01 mg/ml bovine serum albumin (Gibco BRL) dissolved in BEBM.

### 2.3 Real‐time qPCR

Cells were treated with LPS. Real‐time PCR was performed using a BioRad iQ iCycler Detection System (BioRad Laboratories; Hercules) with iQ SYBR Green Supermix. Reactions were performed in a total volume of 20 μl which included 10 μl of 2x SYBR Green PCR Master Mix, 300 nM of each primer and 1 μl of previously reverse‐transcribed cDNA template. The following primers were used: *RIPK3*, forward ACTCCCGGCTTAGAAGGACT, and reverse GCCCTGCTCCTCTTGGTAAG; *IL‐6*, forward CCACACAGACAGCCACTCACC, and reverse CTACATTTGCCGAAGAGCCCTC; *IL‐1β*, forward ACTCCCGGCTTAGAAGGACT, and reverse GCCCTGCTCCTCTTGGTAAG, and β_2_‐microglobulin, used as a reference for normalization, forward CGCTCCGTGGCCTTAGC and reverse GAGTACGCTGGATAGCCTCCA. Real‐time RT‐PCR was performed on a MiniOption Real‐Time PCR Detection System (Bio‐Rad). The parameters were 95°C for 10 min, followed by 40 cycles of 95°C for 15 s, 60°C for 30 s and 72°C for 30 s. All reactions were performed in triplicate. The relative quantity of *mRNA* was obtained using the comparative cycle threshold method and was normalized using β_2_‐microglobulin as an endogenous control.

### 2.4 Transfection and Western Blotting

The plasmid expressing wild‐type RIPK3 was kindly gifted by Dr. You‐Sun Kim (Ajou Univ School of Medicine, Suwon, Korea) and siRNA‐RIPK3 [CUCUCCGCGAAAGGACCAA (dTdT)], and siRNA‐control [CCUACGCCACCAAUUUCGU(dTdT)] were synthesized to Bioneer (Daejeon, Korea). Cells were plated in 6‐well plates 1 day prior to transfection using FuGENE6 (Roche; Indianapolis, IN) according to the manufacturer's instruction. The cells were grown to confluence in 6‐well plates. After treatment with LPS, cells were lysed with 2x lysis buffer (250 mM Tris‐Cl [pH 6.5], 2% SDS, 4% β‐mercaptoethanol, 0.02% bromophenol blue and 10% glycerol). Equal amounts of whole cell lysates were resolved by 8%–10% SDS‐PAGE and transferred to specific membranes. Membranes were blocked with 5% skim milk in Tris‐buffered saline (50 mM Tris‐Cl [pH 7.5] and 150 mM NaCl) for 1 h at room temperature. Blots were then incubated overnight with anti‐phospho‐RIPK3 (Phospho S227; Abcam, ab209384), phospho‐Ser/Thr Phe antibody (Cell Signalling Technology #9631) or total RIPK3 antibody (Cell Signalling Technology #13526) in TTBS (0.5% Tween 20 in Tris‐buffered saline). After washing with TTBS, the blots were further incubated for 45 min at room temperature with anti‐rabbit or anti‐mouse antibody in TTBS and visualized using the ECL system.

### 2.5 F‐Actin staining

F‐actin staining was performed using ActinRed 555 ReadyProbe reagent (Molecular Probes; R37112) following the manufacturer's instructions. The cells were incubated for 30 min, the stain solution removed, and the cells rinsed with PBS. Images were obtained using a Nikon Eclipse 80i microscope (Eclipse 80i) with a 540 nm excitation filter and a 565 nm emission filter.

### 2.6 Trans‐epithelial electrical resistance (TEER)

Before evaluation, the electrodes were sterilized and corrected according to the manufacturer's instructions (Merck). The shorter tip was placed in the culture plate insert, and the longer tip was placed in the outer well. The unit area resistance (Ω × cm^2^) was calculated by multiplying the sample resistance (Ω) by the effective area of the membrane (4.2 cm^2^ for 6‐well Millicell inserts).

### 2.7 LC‐mass spectrometry (MS)/MS for peptides analysis Q‐TOF

#### 2.7.1 Nano‐LC–MS/MS analysis

Nano‐LC–MS/MS analysis was performed with a nano HPLC system (Agilent, Wilmington, DE). A nano‐chip column (Agilent, 150 mm × 0.075 mm) was used for peptide separation. The mobile phase A for LC separation was 0.1% formic acid in deionized water, and the mobile phase B was 0.1% formic acid in acetonitrile. The chromatography gradient was designed for a linear increase from 3% B to 45% B in 30 min, 45% B to 95% B in 1 min, 95% B in 4 min and 3% B in 10 min. The flow rate was maintained at 300 nl/min. Product ion spectra were collected in the information‐dependent acquisition mode and were analysed by Agilent 6530 Accurate‐Mass Q‐TOF using continuous cycles of one full scan TOF MS from 300–2000 m/z (1.0 s) plus three product ion scans from 150–2000 m/z (1.5 s each). Precursor m/z values were selected starting with the most intense ion, using a selection quadrupole resolution of 3 Da. The rolling collision energy feature was used, which determines collision energy based on the precursor value and charge state. The dynamic exclusion time for precursor ion m/z values was 60 s.

#### 2.7.2 Database searching

The mascot algorithm (Matrixscience) was used to identify peptide sequences present in a protein sequence database. Database search criteria were as follows: taxonomy, Homo sapiens; fixed modification, carbamidomethylated at cysteine residues; variable modification, oxidized at methionine residues; maximum allowed missed cleavage, 2; MS tolerance, 100 ppm; and MS/MS tolerance, 0.1 Da. Only peptides resulting from trypsin digests were considered. The peptides were filtered with a significance threshold of *p* < 0.05.

### 2.8 Immunoprecipitation

The cells were harvested by scraping into lysis buffer [25 mM HEPES, 150 mM NaCl, 0.5% NP‐40, 1 mM EDTA, 1 mM EGTA, and protease inhibitor tablet (Complete Mini; Roche)], sonicated (4 times each for 5 s) and centrifuged at 12,000 *g* for 15 min. Supernatant lysates were pre‐cleared with Dynabeads Protein G (Life Technologies) for 30 min at 4°C. Following centrifugation, appropriate antisera were added to the pre‐cleared lysates, incubated for 30 min at 4°C and added Dynabeads Protein G. Immunoprecipitated protein complexes were recovered using DynaMag‐2.

### 2.9 Immunocytochemistry

The cells were fixed with 4% paraformaldehyde, permeabilized with 0.5% Triton X‐100 for 5 min, blocked with 5% BSA for 1 h, labelled with diluted (1:100) anti‐RIPK3 antibody and incubated at 4°C overnight. The secondary antibody, Alexa Fluor 546, was diluted 1:100 in PBS containing 5% BSA and incubated for 2 h at room temperature. Nuclei were stained with diluted Deep‐Red (1:300).

### 2.10 Statistical analyses

The data are presented as the mean ± SD of at least three independent experiments. Where appropriate, statistical differences were assessed by the Wilcoxon–Mann–Whitney test. *p*‐Values < 0.05 were considered statistically significant.

## 3 RESULTS

### 3.1 RIPK3 expression is decreased in inflammation‐related human lung diseases

**FIGURE 1:**
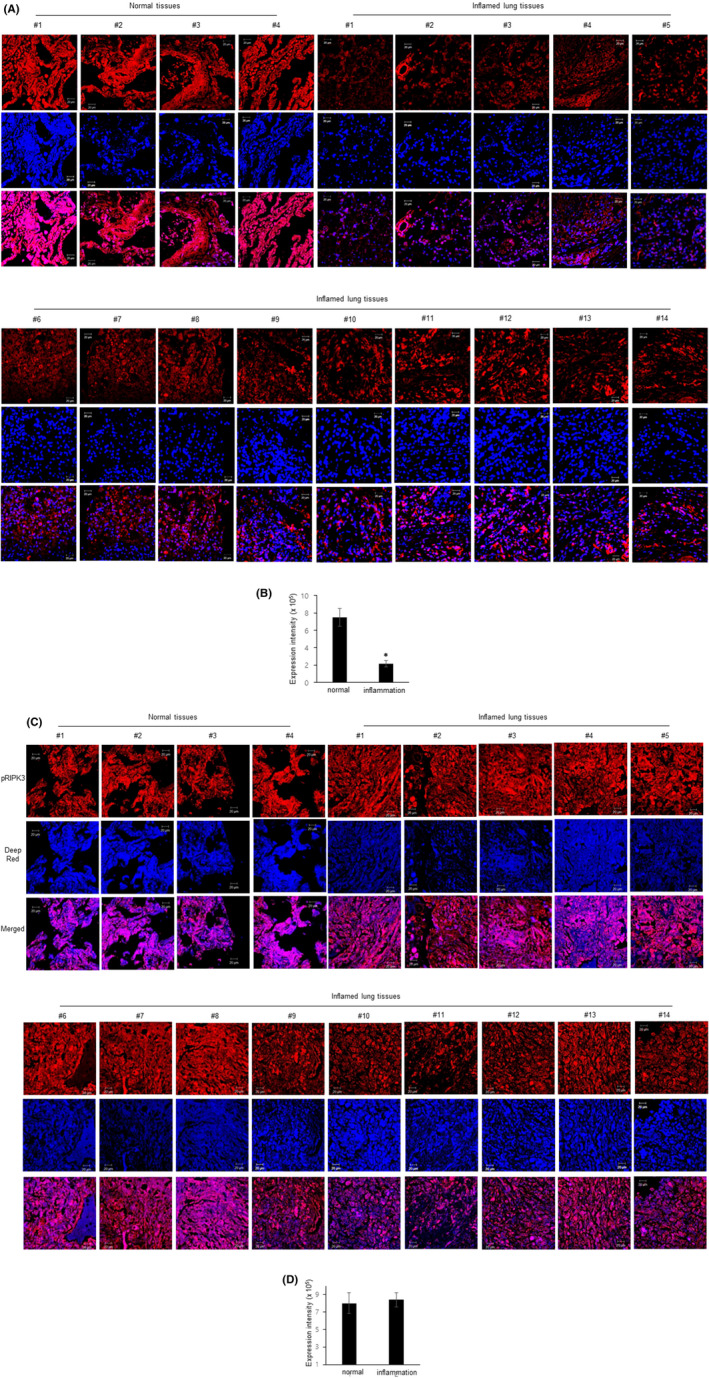
RIPK3 expression in human pulmonary diseases related to inflammation. (A) Two different tissues from normal and inflammation groups were used. Lung sections were processed with anti‐RIPK3 (A) or phospho‐RIPK3 (C) antibody, stained with Deep‐Red, and images were obtained for each core. Five separate images of each section were given a score^1^, ^2^, ^3^, ^4^, ^5^, ^6^, ^7^, ^8^, ^9^, ^10^ by three independent scientists. (B, D) The expression density was used to assess stained differences between cells. *p < 0.05 compared with normal. All data shown are representative of three independent experiments.

To examine whether RIPK3 expression is clinically related to inflammation in human lung tissue, the immunohistochemistry was performed with RIPK3‐specific antibody in human pathological lung tissue array (Figure 1A). Whereas RIPK3 expression was strongly observed in normal tissues, it was decreased dramatically in the inflammation group. Interestingly, RIPK3 expression was detectable in both nuclear and cytoplasm in the normal tissue group, but decreased RIPK3 expression was observed mostly in the cytoplasm of the inflammation group. Interestingly, there was no difference in the level of phospho‐RIPK3 in normal tissue or patient tissue (Figure 1C). Representative images are shown with disease quantification scores (Figure 1B,D). Lung pathologies suggest that a dramatic decrease in RIPK3 expression is closely associated with inflammatory disease processes.

### 3.2 Overexpression of RIPK3 attenuates LPS‐induced airway inflammation in BEAS‐2B bronchial epithelial cells

**FIGURE 2:**
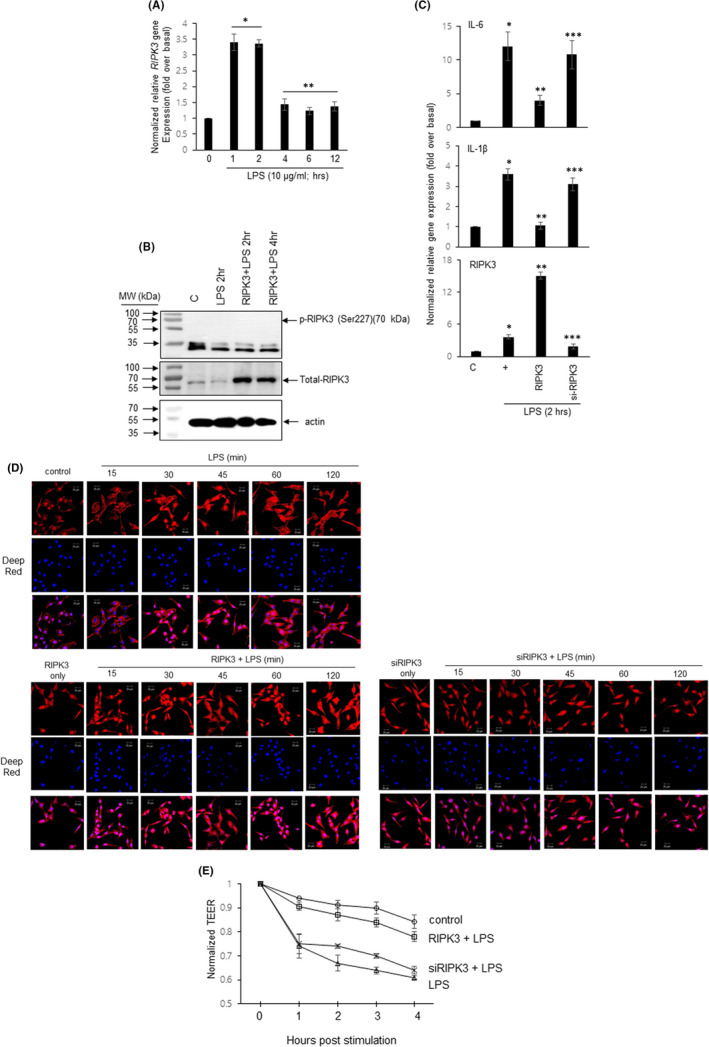
Overexpression of the RIPK3 protein attenuates LPS‐induced F‐Actin formation and *pro‐inflammatory cytokines* gene expression. (A) BEAS‐2b were treated with LPS (10 μg/ml) in a time‐dependent manner (0 ~ 12 h). RIPK3 transcripts were assessed by qRT‐PCR. *p < 0.05 compared to the control, **p < 0.05 compared to LPS for 1 h. (B) BEAS‐2b cells were transfected with wild‐type RIPK3 construct and were then incubated with LPS for 2 ~ 4 h before the generation of total cell lysates, and the phosphorylation of RIPK3 was analysed by Western blotting (C) BEAS‐2b cells were transfected with either wild‐type RIPK3 or siRNA‐RIPK3 construct and were then incubated with LPS for 2 h before the generation of total cell lysates, and pro‐inflammatory cytokine transcripts were assessed by qRT‐PCR. *p < 0.05 compared to the control, **p < 0.05 compared to LPS only, and ***p < 0.05 compared to RIPK3‐transfected cells. (D) Cells were transfected with either a construct driving the expression of wild‐type RIPK3 or RIPK3‐specific siRNA. Cells were then incubated with LPS for 2 h. The cells were stained with ActinRED 555 reagent. Nuclei were stained with diluted Deep‐Red (1:300). (E) After transfection, the cells were treated with LPS in a time‐dependent manner, and then, TEER testing was performed. Error bars represent the mean ± SD of at least three independent experiments. All the data shown are representative of three independent experiments.

To examine whether LPS could control *RIPK3* gene expression at an early event, BEAS‐2B cells were stimulated with LPS in a time‐dependent manner (Figure 2A). *RIPK3* gene expression was diminished at 4 h after LPS treatment. To detect whether LPS induces RIPK3 phosphorylation to activate its own physiological functions, phospho‐antibodies were used. RIPK3 had not been phosphorylated after the treatment with LPS for 2 ~ 4 h using phospho‐Ser277 antibody as well as phospho‐Ser/Thr pan antibody (Figure 2B), suggesting that RIPK3 plays an independent role by a different mechanism in normal human bronchial epithelial cells without phosphorylation by LPS. In addition, to know whether overexpressed RIPK3 expression could regulate the LPS‐induced inflammatory microenvironment and F‐actin formation in bronchial cells, we used specific siRNA‐RIPK3 (Figure 2C). Overexpressed RIPK3 could significantly decrease pro‐inflammatory cytokine gene expression (IL‐6 and IL‐1β); however, siRNA‐RIPK3 did not affect the expression of these genes. More interestingly, with LPS only, F‐actin was generated throughout the cell at 30–45 min. Whereas F‐actin was continuously and strongly generated throughout the cell for 120 min with RIPK3 overexpressed, it was not increased by LPS in the cells transfected with siRNA‐RIPK3, and, apical extension and protrusion were dramatically decreased in the cells transfected with siRNA‐RIPK3 (Figure 2D). In addition, we found that even trans‐epithelial electrical resistance (TEER) was reinstated by the overexpression of RIPK3, but not by siRNA‐RIPK3 (Figure 2E). These results suggest that overexpression of RIPK3 could change the inflammatory microenvironment after LPS treatment in human bronchial epithelial cells.

### 3.3 RIPK3 bound to ubiquitin protein regulates RIPK3 functioning after LPS treatment

**FIGURE 3:**
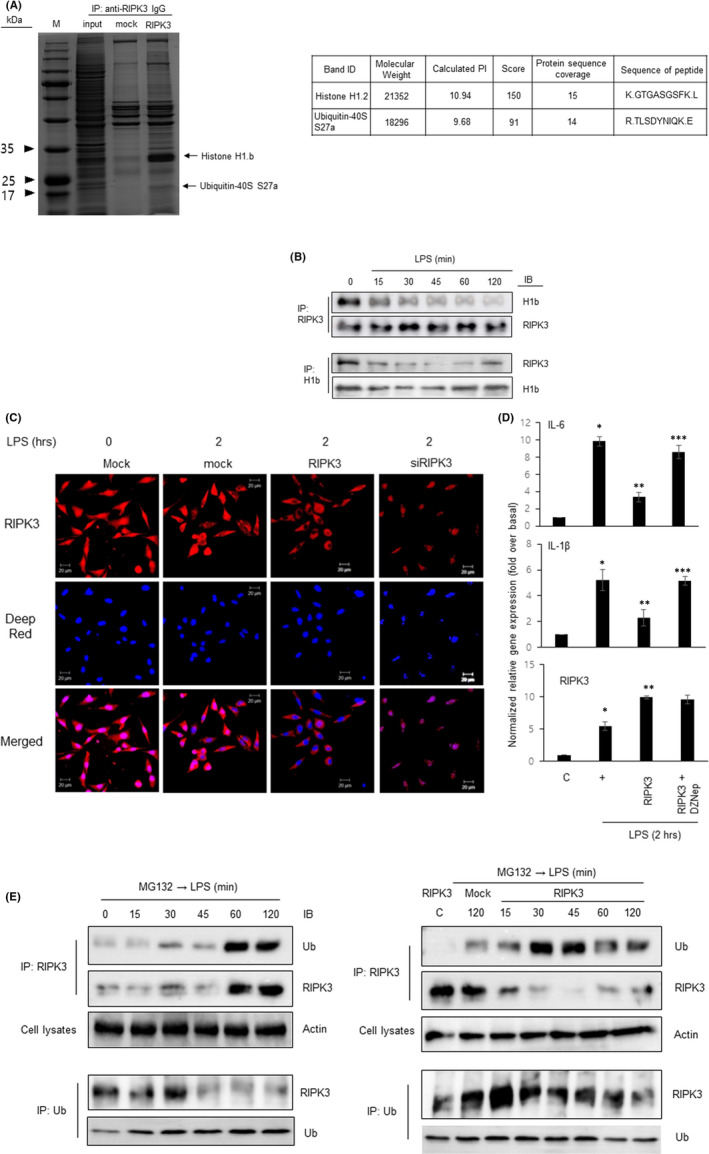
Histone methylation dissociates the interaction with RIPK3 and RIPK3 translocates to the cytoplasm. (A) The cells transfected with either pcDNA3 (mock) or wild‐type RIPK3 were harvested; total cell lysates were then immunoprecipitated with anti‐RIPK3 antibody and were resolved by SDS–PAGE. The gel was stained with Coomassie‐blue, and nano‐LC analysis was performed. Information of the specific band identified which is marked by the arrow on the right is summarized. The input (from *pcDNA3.1::RIPK3* Transfectants) lane contains one‐tenth of the lysate volume used for immunoprecipitation. (B) The cells were treated for indicated times with LPS. Total cell lysates were immunoprecipitated with anti‐RIPK3 antibody (upper panel) or anti‐Histone antibody (lower panel) and blotted with anti‐H1b (upper) or anti‐RIPK3 (lower) antibody. The same membrane was stripped and reprobed with anti‐RIPK3 or anti‐Histone antibody as an input control. IB, immunoblotting; IP, immunoprecipitation. (C) The cells were incubated on a chamber slide, fixed with 4% paraformaldehyde and processed for immunofluorescence with RIPK3 and Alexa Fluor 546 antibodies. The nuclei were stained with Deep‐Red. The scale bar represents 20 μm. (D) BEAS‐2B cells were transfected with either wild‐type or siRNA‐RIPK3 construct, and then incubated with 0.5 μM of DZNep, histone methylation inhibitor, for 72 h before the treatment of LPS, and pro‐inflammatory cytokine transcripts were assessed by qRT‐PCR. *p < 0.05 compared to the control, **p < 0.05 compared to LPS only, and ***p < 0.05 compared to RIPK3‐transfected cells. All the data shown are representative of three independent experiments. (E) Confluent (upper panel) cells or cells transfected with wild‐type RIPK3 (lower panel) were treated with 20 μM of MG132 for 4 h before treatment with LPS for the indicated times. Total cell lysates were then immunoprecipitated with anti‐RIPK3 antibody and blotted with anti‐ubiquitin antibody. IB, Immunoblotting; IP, Immunoprecipitation. Actin expression was used as a loading control. The figures are representative of three independent experiments.

Since RIPK3 is a multifunctional signalling mediator, we thought it might combine with a protein complex to apply its potential roles. To investigate RIPK3‐binding partners in mammalian cells, we immunoprecipitated the RIPK3 complex from BEAS‐2B cells transfected with the RIPK3 construct (Figure 3A). Coomassie‐blue staining detected a unique protein band in the RIPK3‐immune complex. The RIPK3‐specific band was analysed by LC–MS and identified as histone H1.2 and Ubiquin‐40S S27a protein (Figure 3A). Immunoprecipitation of the lysates was used to examine the association between endogenous H1B and RIPK3 and showed that RIPK3 was bound to H1B. Surprisingly, this interaction was dissociated after stimulation with LPS for 15 min (Figure 3B, upper panel). Similarly, anti‐Histone antibody efficiently immunoprecipitated Histone from cell lysates and also coimmunoprecipitated RIPK3 (Figure 3B, lower panel). RIPK3 protein (upper panel) or Hib protein (lower panel) level itself as an input control was not decreased under LPS‐incubation at these time points. These results suggest that histone 1B was bound to RIPK3 in the nucleus to prevent translocation towards the cytoplasm before LPS stimulation. Thus, we performed immunocytochemistry and analysed RIPK3 expression levels in the nuclear and cytoplasmic regions of BEAS‐2B cells with/without LPS stimulation (Figure 3C). Interestingly, RIPK3 expression was observed mainly in the nuclear region without LPS treatment, whereas it strongly translocated to the cytoplasmic region after LPS treatment, but not siRNA‐RIPK3 (Figure 3C). We performed the Western blot analysis with nuclear and cytosol fractionation to confirm the results of immunocytochemical staining. CREB was used as a fractionation control. We found RIPK3 expression was decreased in nuclear fraction, whereas it was slightly increased in cytosolic fraction after LPS treatment (Figure S1). In addition, to know which physiological mechanism disintegrated the interaction between H1B and RIPK3, we used a histone deacetylase inhibitor (SAHA) or an inhibitor of histone methyltransferase EZH2 (DZNep). SAHA (2 μM) was treated for 12 h or DZNep (0.5 μM) was treated for 72 h before LPS stimulation. The gene expression of pro‐inflammatory cytokines was significantly increased after DZNep (Figure 3D), but not SAHA (data not shown), indicating because the histone was methylated, it could not no longer be combined with RIPK3, and RIPK3 was translocated to the cytoplasm in an anti‐inflammatory function. According to the results of the LC–MS/MS (Figure 3A), RIPK3 was bound to ubiquitin without LPS stimulation. To understand the physiological significance of this interaction between RIPK3 and ubiquitin, an immunoprecipitation assay was performed (Figure 3E). RIPK3 ubiquitination by LPS reached a peak at 60 min and was maintained at 120 min. In addition, the expression of RIPK3 was the same as the previous result (Figure 2A). Interestingly, RIPK3 ubiquitination by LPS reached a peak at 60 min and gradually declined at 120 min in the cells transfected with the wild‐type RIPK3 construct, and the expression of RIPK3 was quickly degraded at 30 min after LPS treatment (Figure 3E, upper panel). Conversely, when immunoprecipitation assay was performed, ubiquitin bound rapidly, and then, ubiquitination has been processed. After 60 min, the binding to RIPK3 seemed weak (Figure 3E, lower panel). We propose that the anti‐inflammatory signalling of RIPK3 inhibits the inflammation signalling by LPS over a short time and then degrades, the signal of the inflammatory response by LPS is transmitted, and the ubiquitination of endogenous RIPK3 occurs at a later time.

### 3.4 RIPK3 inhibits pro‐inflammatory cytokine gene expression by inhibiting the NF‐kB pathway activated by LPS

**FIGURE 4:**
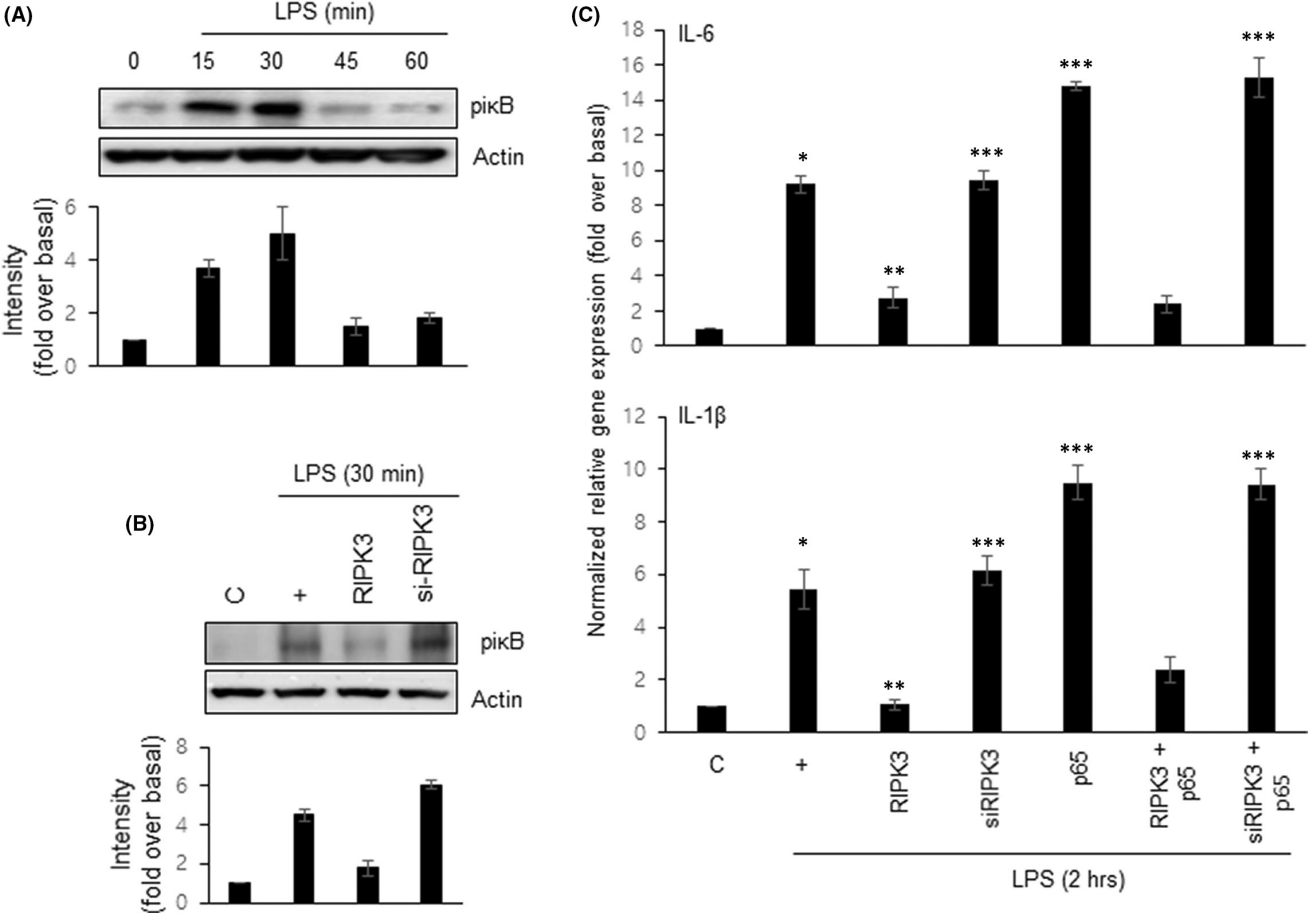
Overexpression of RIPK3 inhibits LPS‐induced NF‐κB activation to diminish pro‐inflammatory cytokine gene expression. (A) BEAS‐2B cells were treated with LPS (10 μg/ml) in a time‐dependent manner (15 ~ 60 min). NF‐κB activation was assessed by Western blotting. (B) The cells were transfected with either wild‐type RIPK3 or siRNA‐RIPKs and were then incubated with LPS for 30 min before the generation of total cell lysates. (C) The cells were transiently transfected with wild‐type or siRNA constructs of RIPK3 or a p65 construct, and cells were then serum‐starved and treated with LPS for 2 h, after which cell lysates were harvested for real‐time PCR. *p < 0.05 compared with control; **p < 0.05 compared with LPS treatment; ***p < 0.05 compared with wild‐type RIPK3 transfectants. All the data shown are representative of three independent experiments.

To determine which signal pathway is activated within the cells stimulated by LPS, we performed Western blot analysis using a phospho‐specific antibody (Figure 4A). Because NF‐κB signalling pathway is essential for LPS signalling, we checked whether LPS‐induced NF‐κB signalling was affected by RIPK3. IκB phosphorylation was maximally activated at 30 min, and this effect decreased at 45 min (Figure 4A). Interestingly, RIPK3 dramatically inhibited LPS‐induced iκB phosphorylation for 30 min, but not siRNA‐RIPK3 (Figure 4B). Furthermore, to determine whether RIPK3 influences LPS‐induced pro‐inflammatory cytokine gene expression through the NF‐κB pathway, we used a p65 overexpression construct, which is a signalling protein downstream of iκB. Overexpression of p65 increased the expression of pro‐inflammatory cytokine genes but cotransfection with both RIPK3 and p65 constructs dramatically decreased expression (Figure 4C), indicating that NF‐κB appears to be controlled by RIPK3.

## 4 DISCUSSION

*P. aeruginosa* is a dominant organism within the hospital environment, an increasingly multidrug‐resistant microorganism, and the most common Gram‐negative pathogen causing hospital‐acquired pneumonia.^19^, ^20^ In the lung, *P. aeruginosa* has been known to opportunistically colonize patients with CF^21^ and chronic obstructive pulmonary disease.^22^ Chronic *P. aeruginosa* infections result from the dynamic and complex interactions between pathogen and host, where bacteria continue without causing overwhelming host injury, and where host defences fail to eliminate the pathogen.^23^ Although various diseases in humans are caused by *P. aeruginosa*, treatment is limited due to drug resistance. This study is focused on the use of intracellular proteins to overcome drug resistance and on providing new therapeutic options to overwhelm *P. aeruginosa*.

RIPK3 is a multifunctional regulator of cell death and inflammation that controls signalling downstream of the tumour necrosis factor receptor family, DNA‐dependent activator of IFN‐regulatory factors and toll‐like receptors (TLRs).^24^ Several reports have shown that RIPK3 was regulated by post‐translational modification and regulatory proteins, such as protein phosphatase 1B (Ppm1b), deubiquitylating enzyme A20, the C terminus of Hsp‐70‐interacting protein (CHIP) or Pellino E3 ubiquitin protein ligase (PELI) 1.^14^ Although it is very important to identify/characterize RIPK3‐binding proteins or the physiological mechanism in the human body, there is no study on the function of RIPK3 in the respiratory tract during *P. aeruginosa* infection. As mentioned above, because infection with *P. aeruginosa* is very severe and harmful in many diseases, we investigated the effect of RIPK3 on *P. aeruginosa*‐induced acute airway inflammation.

Both the expression of RIPK3 and phosphorylated RIPK3 were remarkably decreased in the human inflamed pathological lung group, compared to the normal group (Figure 1). Although phosphorylated RIPK3 promotes the intracellular apoptotic pathway^14^, ^25^ and overexpressed RIPK3 induced MLKL phosphorylation‐mediated programmed necrosis,^26^ it is still unclear why RIPK3 expression, but not phospho‐RIPK3 was dramatically decreased in human pathological lungs, and cell death was not observed in the tissues (Figure 1C). The possibilities are that (1) RIPK3 may act as an anti‐inflammatory regulator in the lung. Why the RIPK3 protein, which is expressed strongly in normal lung tissues, could not be observed in inflammatory tissues could mean that RIPK3 may promote anti‐inflammatory phenomena. We will explain this possibility in more detail later (Figure 2). (2) There will be other unknown proteins that can bind to RIPK3 and affect its physiological function in the human lung. A common feature of the RIPK3‐binding proteins mentioned in the above paragraph is the cytoplasmic location. Interestingly, cytoplasmic proteins could affect post‐translational modifications, but there have been no studies of events occurring in the nucleus and the post‐transcriptional modification so far. We will also explain this possibility in more detail later (Figure 3). Together, the function of RIPK3 is still not known accurately, and unlike the lungs of normal tissue, RIPK3 expression can be dramatically reduced in the inflammatory pathological lung, and more interestingly, the expression of RIPK3 after LPS stimulation translocated towards the cytoplasm, not the nucleus. Taken together, RIPK3 expression was significantly decreased and translocated towards the cytoplasm in human pathological lungs in our system.

To examine how RIPK3 translocated from the nucleus to cytoplasm in the pathological lung or after treatment with LPS (Figure 3), 1D LC–MS/MS analysis was performed. According to several studies, RIPK3 mainly binds to cytoplasmic proteins, but RIPK3 is also expressed in the nucleus. In our system, RIPK3 was bound to the ubiquitin protein (Figure 3A). The protein complex with H1b protein bound to RIPK3 was in the nucleus. However, on stimulation(s), it became an acetylation of H1b that was no longer bound to RIPK3 and dissociated, proving that RIPK3 finally translocated to the cytoplasm and played an independent function in airway cells. Many studies show that gene expression is controlled by acetylation.^27^ However, to our knowledge, there is no study on histone in signalling mechanism or physiological phenomena related to RIPK3, and this study is the first in the respiratory system. Therefore, further research on this should be conducted in‐depth.

RIPK3 is known as a switch protein that determines necroptosis or inflammation. Interestingly, Moriwaki et al.^28^ demonstrated that RIPK3 protects against dextran sodium sulphate (DSS)‐induced colitis and that RIPK3 is required for tissue repair by inducing an axis of IL‐23, IL‐1β and IL‐22 downstream of DSS‐induced injury, independent of its role in cell death. In our system, RIPK3 inhibited the expression of *IL‐1β* and *IL‐6* genes by inhibiting the activity of the iκB pathway, thus finally creating an anti‐inflamed microenvironment (Figure 4C). These differences are caused by differences in the cells, but the main cause is differences in the surrounding environment. Unlike other tissues, the lungs are always in contact with external air, and gas exchange occurs in blood vessels and the alveolar of surrounding tissues. Recently, Han et al.^29^ reported that BEAS‐2B cells have the specific features of mesenchymal stem cells. In that paper, they used α‐MEM complete media with 10% FBS to incubate BEAS‐2B cells. We found that the morphology of BEAS‐2B cells was dramatically changed when the cells were incubated in α‐MEM with 10% FBS (Figure S2). The morphological change seems to be caused by latent TGFβ1, which is already contained at a high level in foetal bovine serum,^30^ based on a recent report that TGFβ1 could induce epithelial to mesenchymal transition in BEAS‐2B cells.^31^ To culture BEAS‐2B cells in this research, we utilized the BEGM TM Bronchial Epithelial Cell Growth Medium Bullet Kit TM (Ronza, CC‐3170), containing hEGF instead of TGFβ1. In our culture circumstances, the observation of morphological change of the cells was not detected at all. This media has been known to be proper to incubate primary bronchial epithelial cells and maintain the properties of epithelial cells.

We found that RIPK3 expression was decreased in human pathological lungs and had an inhibitory effect on LPS‐induced airway inflammation and TEER. In addition, H1B bound to RIPK3 is dissociated by methylation after LPS stimulation. Translocated RIPK3 inhibited iκB activation to suppress the expression of pro‐inflammatory cytokine genes. Thus, these results suggest that RIPK3 may be a potential therapeutic candidate during *P. aeruginosa* infection‐induced respiratory diseases.

## AUTHOR CONTRIBUTIONS

**Minsu Yun:** Conceptualization (equal); data curation (equal); investigation (equal); writing – original draft (equal). **Sun‐Hee Park:** Conceptualization (lead); data curation (lead); formal analysis (lead). **Dong Hee Kang:** Data curation (equal); methodology (equal). **Ji Wook Kim:** Data curation (equal); formal analysis (equal); investigation (equal). **Ju Deok Kim:** Investigation (equal); resources (equal); validation (equal). **Siejeong Ryu:** Supervision (equal); validation (equal). **Jeongyeob Lee:** Formal analysis (equal); validation (equal). **Hye Min Jeong:** Investigation (equal); methodology (equal). **Hye Ran Hwang:** Investigation (equal); methodology (equal). **Kyoung Seob Song:** Conceptualization (equal); validation (equal); writing – original draft (equal).

## FUNDING INFORMATION

This study was supported by a grant from the National Research Foundation of Korea (NRF) and funded by the Korean government (NRF‐2020R1I1A2075001 and NRF‐2021R1A4A1031380 to K.S.S.).

## CONFLICT OF INTEREST

We declare that we do not have any conflicts of interest.

## Supporting information


Figure S1

Figure S2

Figure Legends S1‐S2

## Data Availability

The data that support the findings of this study are available from the corresponding author upon reasonable request.
