# Supplementary figures and images for: Inhibition of Pseudomonas aeruginosa LPS‐Induced airway inflammation by RIPK3 in human airway
Source: J Cell Mol Med. 2022 Oct 13;26(21):5506–16. doi: 10.1111/jcmm.17579 (PMC9639037; doi:10.1111/jcmm.17579)

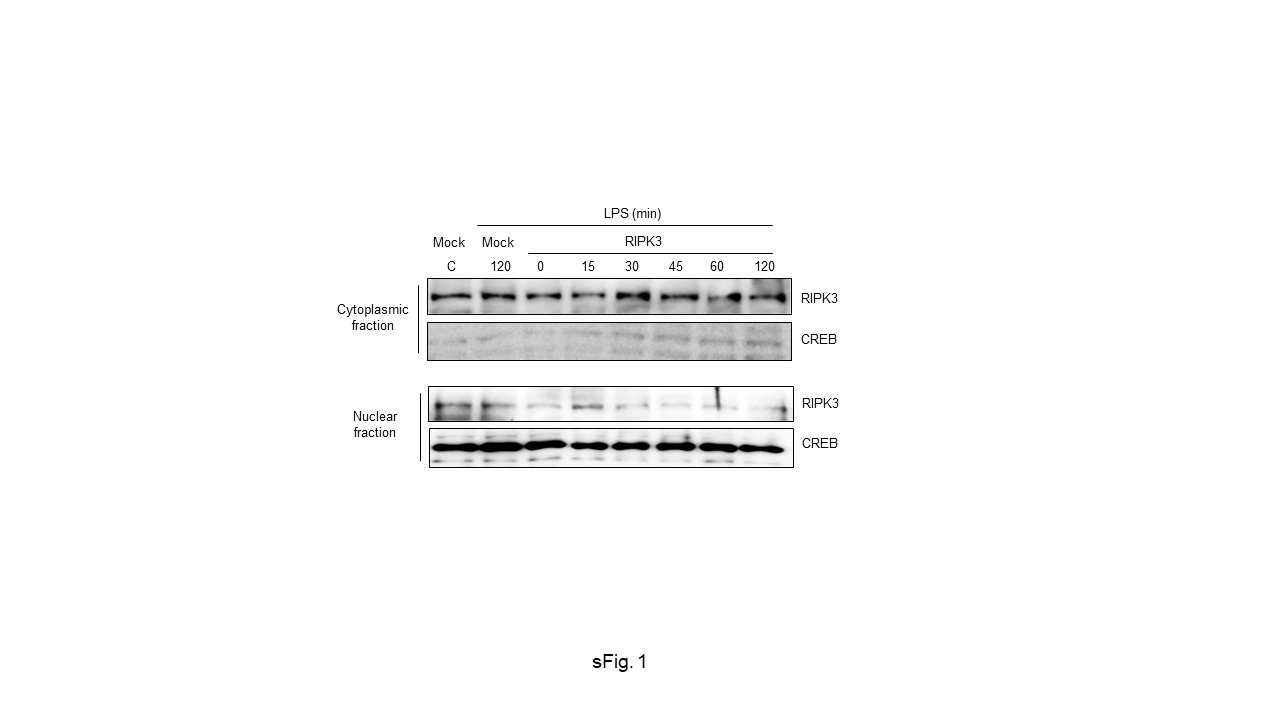

Supplement: Supplementary file 1 — Figure S1 [file JCMM-26-5506-s003.tif]

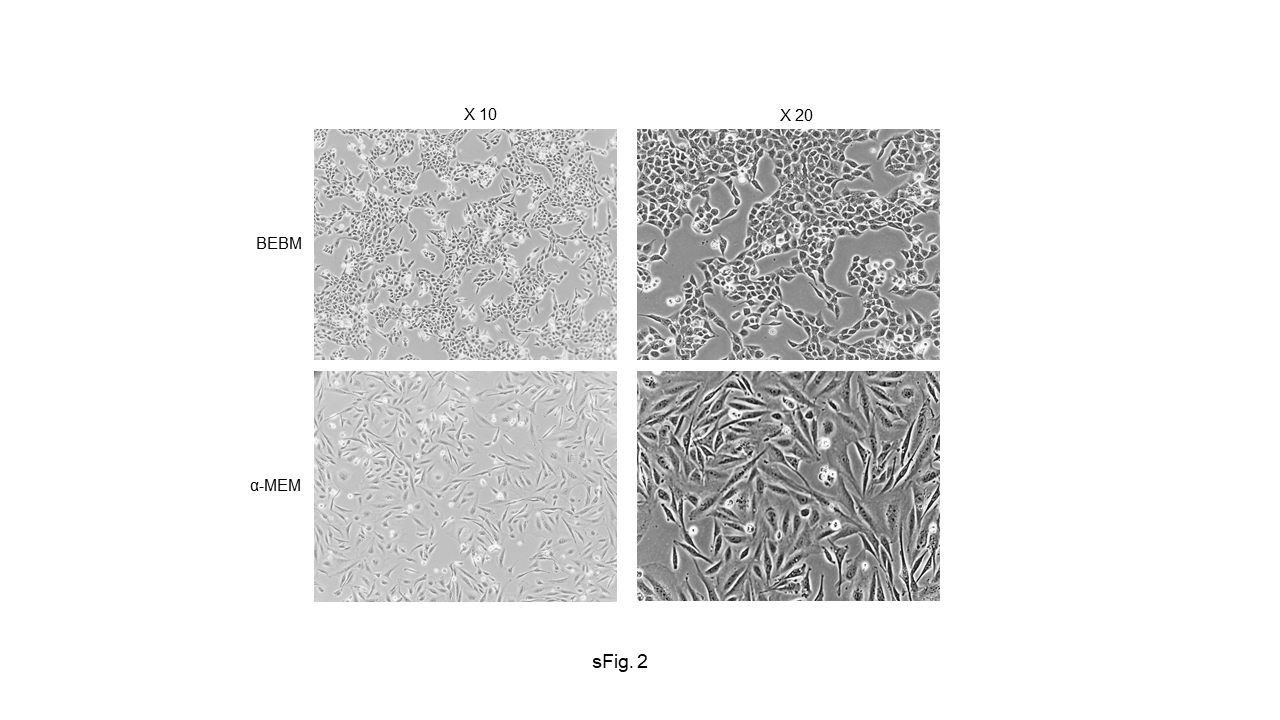

Supplement: Supplementary file 2 — Figure S2 [file JCMM-26-5506-s001.tif]
