# Supplementary material for: Inhibition of Pseudomonas aeruginosa LPS‐Induced airway inflammation by RIPK3 in human airway
Source: J Cell Mol Med. 2022 Oct 13;26(21):5506–16. doi: 10.1111/jcmm.17579 (PMC9639037; doi:10.1111/jcmm.17579)
Supplement: Supplementary file 3 — Figure Legends S1‐S2 [file JCMM-26-5506-s002.docx]

Supplementary Figure 1. RIPK3 expression translocated from nucleus to cytoplasm after LPS treatment. LPS was treated in a time-dependent manner in the cells transfected with RIPK3 construct, and then the fractionation assay was performed using NE-PER™ Nuclear and Cytoplasmic Extraction Reagents (Thermo Fisher, #78833). Nuclear CREB expression was used as a fractionation control.

Supplementary Figure 2. α-MEM media containing 10% FBS induces morphological change. Normal human bronchial epithelial (BEAS-2b) cells were cultured in BEBM (Lonza) with a BEGM kit at 37 °C in a humidified incubator with 5% CO_2_. In addition, the cells were α-MEM media containing 10% FBS for 5 days. The images were obtained using a Nikon Eclipse 80i microscope (Eclipse 80i) were taken he images were taken at magnifications of X10 and X20.
